# Complete Genome Sequence of the Sugar Beet Endophyte *Pseudomonas poae* RE*1-1-14, a Disease-Suppressive Bacterium

**DOI:** 10.1128/genomeA.00020-13

**Published:** 2013-03-07

**Authors:** Henry Müller, Christin Zachow, Mohammadali Alavi, Ralf Tilcher, Peter Mauritius Krempl, Gerhard Günther Thallinger, Gabriele Berg

**Affiliations:** a Institute of Environmental Biotechnology, Graz University of Technology, Graz, Austria; b Austrian Centre of Industrial Biotechnology (ACIB GmbH), Graz, Austria; c KWS SAAT AG, Einbeck, Germany; d Institute for Genomics and Bioinformatics, Graz University of Technology, Graz, Austria

## Abstract

The endophytic bacterium *Pseudomonas poae* RE*1-1-14 shows broad antagonistic activity and is applied to seeds as a biocontrol agent to suppress late root rot in the sugar beet. The completely sequenced 5.5-Mb genome reveals genes that putatively contribute to this antagonistic activity and the intimate plant-microbe interaction.

## GENOME ANNOUNCEMENT

*Pseudomonas poae* RE*1-1-14 is a member of the group of pseudomonads that interact beneficially with plants. Based on the 16S rRNA gene sequence, *P. poae* RE*1-1-14 is closely related to strains *Pseudomonas trivialis* DSM 14937^T^ and *P. poae* DSM 14936^T^, distinguished by their ability to utilize sucrose (1). RE*1-1-14 was isolated from the endorhiza of the sugar beet and exhibits antagonistic activity against the phytopathogens *Phoma betae, Rhizoctonia solani* AG2-2IIIB, *R. solani* AG4, and *Sclerotium rolfsii* (2). Reintroduced to sugar beet seeds, *P. poae* RE*1-1-14 was demonstrated to densely colonize emerging roots, a primary requirement for the effective suppression of root pathogens (2). In field trials performed over six consecutive years, this isolate was proven to control late root rot caused by *R. solani* (unpublished data).

The genome of *P. poae* RE*1-1-14 was sequenced using a combination of next-generation sequencing platforms. A first-draft assembly based on 882,576 reads of an 8-kbp paired-end library (Roche 454 GS FLX Titanium) (Center for Medical Research [ZMF], Medical University of Graz, Austria) with a total of 171.1 Mb (31-fold coverage) was generated with Newbler 2.6 (Roche Diagnostics, Penzberg, Germany). This assembly consisted of 144 contigs, 66 of which could be joined into a single circular scaffold. Gaps resulting from repetitive sequences were resolved by *in silico* gap filling, and the remaining gaps were closed by PCR followed by Sanger sequencing, yielding a draft genome of 5,512,225 bp. To improve the quality of the sequence by eliminating 454 sequencing artifacts in homopolymer stretches, the genome was subsequently sequenced using the Illumina paired-end method (Illumina HiSeq 2000; Ambry Genetics, Aliso Viejo, CA) (6,973,734 reads, 697 Mb; 128-fold coverage). The Illumina reads were aligned to the draft genome with CLC Genomics workbench 4.7.2 (CLC bio, Aarhus, Denmark). The final consensus sequence was derived by counting the instances of each nucleotide at a particular position and then letting the majority decide the nucleotide for the consensus sequence.

Genes were identified with the Prodigal gene finder (3), ARAGORN (4), and RNAmmer 1.2 (5). Functional annotation of the predicted genes was performed using BASys (6), which provides annotations with respect to the Clusters of Orthologous Groups (COG) (7), Pfam (8), and Gene Ontology (GO) (9) databases. The final genome includes 5,512,241 bases, with a G+C content of 60.85%. The number of putative genes totals 4,854, of which 4,768 are protein coding. There are five instances of the ribosomal 5S-23S-16S cluster, an additional 5S rRNA gene, and 70 tRNAs. *P. poae* RE*1-1-14 additionally harbors a plasmid consisting of 6,375 bp and carrying 15 putative genes.

A cursory search of the genome sequence revealed the presence of gene clusters putatively involved in antagonistic activity, including genes for the synthesis and exudation of hydrolytic exoenzymes and cyclic lipopeptides. Various types of secretion systems and genes encoding the 1-aminocyclopropane-1-carboxylate deaminase indicate an ability to interact closely with plants. Despite its somewhat high similarities to the genomes of other sequenced plant-associated *Pseudomonas* strains, *P. poae* RE*1-1-14 possesses a genome with a unique assemblage of accessory genes.

### Nucleotide sequence accession number.

The genome sequence for *P. poae* RE*1-1-14 has been deposited at EMBL/GenBank under the accession no. CP004045.
